# A rare case of pure testosterone-secreting adrenal adenoma in a postmenopausal elderly woman

**DOI:** 10.1186/s12902-019-0342-y

**Published:** 2019-01-23

**Authors:** Wei-bin Zhou, Nan Chen, Cheng-jiang Li

**Affiliations:** Department of Endocrinology, The First Affiliated Hospital, College of Medicine, Zhejiang University, Zhejiang, 310003 Hangzhou China

**Keywords:** Hyperandrogenism, Postmenopause, Adrenal tumors, Testosterone, Immunohistochemistry

## Abstract

**Background:**

Hyperandrogenemia is more common in puberty and reproductive age, but relatively rare in postmenopausal women. Postmenopausal virilization may result from androgen-producing tumors. Androgen-secreting adrenal tumors are rare in clinical practice and are diagnosed as adrenocortical carcinoma, most of which can co-secrete androgen and cortisol. Highly elevated serum testosterone level with normal adrenal androgens such as dehydroepiandrosterone (DHEA), dehydroepiandrosterone sulfate (DHEAS) and androstenedione is usually regarded as ovary origin. Here we describe an unusual case of a postmenopausal woman with markedly elevated serum testosterone level, while DHEAS, androstenedione, 17-hydroxyprogesterone and cortisol were within the normal range.

**Case presentation:**

A 67-year-old postmenopausal woman with hirsutism in the upper lip and armpit, accompanied by clitoromegaly for 5 months. Hormonal evaluation showed markedly elevated serum testosterone level (714.8 ng/ml), whereas DHEAS, androstenedione, 17-hydroxyprogesterone, and cortisol were within the normal range. Imaging examination showed a mass of 1.5 cm in diameter in the left adrenal gland and normal appearance of both ovaries. PET-CT indicated that it was a case of benign adrenal adenoma and excluded ovarian abnormalities and other ectopic tumors. Thus, a pure testosterone-secreting adrenal tumor was suspected and then adrenalectomy was performed. Histology and immunohistochemistry furtherly confirmed the benign adrenocortical adenoma with immunohistochemistry positive for inhibin α, melan A, β-captenin, SYN (focal), Ki-67(< 3%), and negative for chromogranin (CgA), cytokeratin (CK), S-100, P53. After surgery, the level of testosterone returned to normal range and the clinical symptoms also subsided.

**Conclusions:**

Pure testosterone-secreting adrenal adenomas are extremely rare, but it can induce severe hyperandrogenism and virilization. The source identification of hyperandrogenemia only based on the levels of testosterone, DHEAS and androstenedione is limited. It is important to evaluate not only ovaries but also adrenals in all women with virilization particularly during menopause, even their androstenedione, DHEA and DHEAS level are normal.

## Background

Hyperandrogenemia is more common in adolescence and childbearing age, but relatively rare in postmenopausal women. Due to the lack of clinical and epidemiological data of postmenopausal women with hyperandrogenemia, the clinical diagnosis and treatment are often difficult. A highly elevated testosterone level and manifestations of hyperandrogenism such as hirsutism and virilization in a postmenopausal woman strongly suggest the potential androgen-producing tumor [1]. Dehydroepiandrosterone (DHEA), dehydroepiandrosterone sulfate (DHEAS) and androstenedione are mainly produced in the adrenal glands. The increased DHEAS was used as a marker of increased adrenal activity, so the circulating DHEAS level has been used to screen androgen from ovary or adrenal gland [2]. Functional adrenal tumors mainly include those secreting aldosterone or cortisol, and a few secreting both cortisol and androgen, but pure androgen-secreting adrenal tumors (PASATs) are rare, most of which are reported as case studies, and this kind of PASATs that only secrete testosterone with normal adrenal androgens (DHEA, DHEAS and androstenedione) are more rare [3]. Herein, we report a case of benign androgen-secreting adrenal tumor in a postmenopausal elderly woman, with markedly elevated testosterone levels while DHEAS and androstenedione were within normal range.

## Case presentation

A 67-year-old postmenopausal woman with hirsutism of increased hair around the upper lip and armpit and clitoromegaly for five months was referred to the endocrinology clinic of our hospital. She had normal physiological development during her infancy and childhood, and also has a normal sexual life with no other medical history. Her menarche was at 18 years old, and her menopause at age of 56. She had a normal menstrual history before menopause and had no postmenopausal bleeding. She had two healthy children and no miscarriages. She denied taking estrogen, progesterone or health care products. There are no similar patients in her family.

On physical examination, she was 153 cm tall and weighed 53 kg with body mass index of 22.6 kg/m^2^. Increased hair was observed in her upper lip and armpit (Ferriman- Gallwey score of 8), and a physical examination of genital revealed clitoromegaly. There was no acne, deepening of the voice or other virilization signs. Findings on examination of the head and neck, breasts and abdomen were unremarkable. She had no signs of Cushing syndrome, or acanthosis nigricans syndrome.

The hormonal test showed high total testosterone levels (714.8 ng/dL, reference value 14–56). Serum DHEAS (145.8 ng/mL, reference value 25.9–460.2), androstenedione (2.4 ng/mL, reference value 0.3–3.3) and 17-hydroxyprogesterone (1.7 nmol/l, reference value 0–11.5) levels were within normal range. The serum values of follicle-stimulating hormone, luteinizing hormone, and prolactin were also within the normal range for the menopause. The levels of anti-mullerian hormone, human chorionic gonadotropin (hCG), thyroid- stimulating hormone (TSH), plasma renin activity and aldosterone, adrenocorticotropic hormone (ACTH), serum cortisol, 24-h urinary free cortisol, and 1 mg dexamethasone suppression test were in normal range. The ovarian tumor markers (Ca 125, CEA, Ca 199) were in normal reference range. The repeated samples confirmed that her high testosterone levels were within the tumor range. We excluded overt Cushing Syndrome on the basis of normal cortisol suppression after 1 mg dexamethasone and normal urinary free cortisol levels, as recently proposed by Ceccato F [4]. Then a middle dosage dexamethasone test (0.75 mg, 4 times a day for 5 consecutive days) without testosterone inhibition strongly suggested the potential androgen-producing tumor, further examinations were needed to distinguish ovarian or adrenal origin of hyperandrogenemia.

**Fig. 1:**
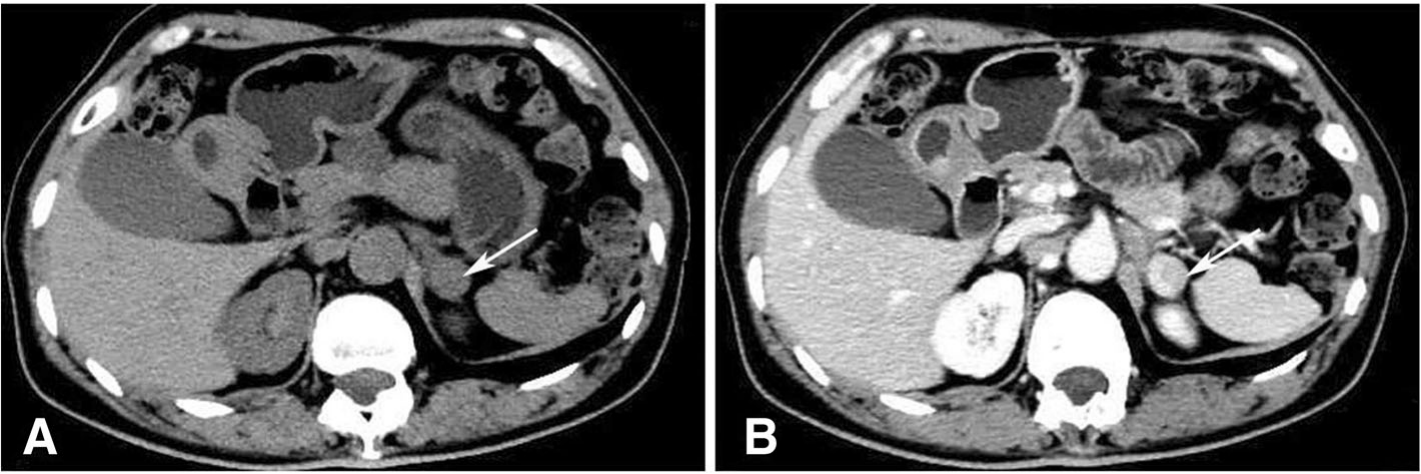
Adrenal CT (**a**) and enhanced CT (**b**) identified a left adrenal mass, 1.5 cm in diameter (Arrow). CT = computed tomography

Initially, the lack of co-secretion of DHEAS and androstenedione indicated that her elevated testosterone might be of ovarian origin. However, pelvic ultrasound disclosed that there was no ovarian mass, while adrenal ultrasound showed a hypoechoic nodule in the left adrenal gland. Further pelvic magnetic resonance image (MRI) showed submucous myoma of uterus, but no abnormal of ovarian, and adrenal CT scan was also performed and a left adrenal mass of about 1.5 cm in diameter was revealed (Fig. 1 a and b). PET-CT confirmed a round nodule in the external branch of the left adrenal gland with slight increase in FDG metabolism (the SUV max of the nodule was 2.56), considering the possibility of benign adenoma. No ovarian abnormalities or other ectopic tumors were found by PET-CT.

**Fig. 2:**
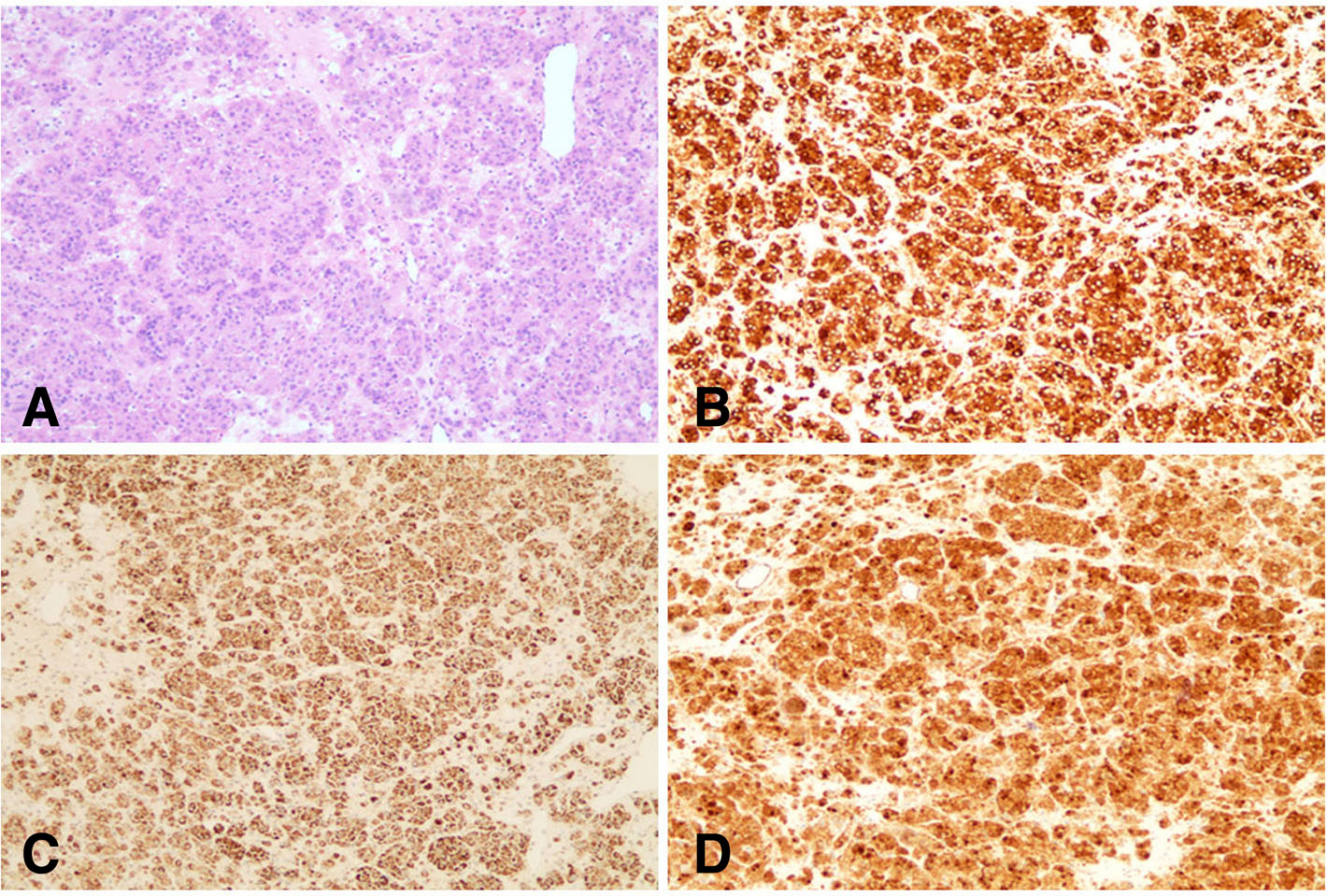
Hematoxylin and eosin staining of the adrenal tumor. Magnification× 100 (**a**); immunohistochemistry showing tumor tissue that stained positive for inhibin α (**b**), melan A (**c**), β-captenin (**d**)

Based on the clinical characteristics, hormone detection and imaging appearances of the case, pure testosterone-secreting adrenal tumor was suspected. Subsequently, the patient underwent a laparoscopic resection of left adrenal tumor. Histological examination (Fig. 2 a) and immunohistochemistry also confirmed the diagnosis of benign adrenocortical adenoma with immunohistochemistry positive for inhibin α, melan A, β-captenin (Fig. 2 b-d), SYN (focal), Ki-67 (< 3%), and negative for chromogranin (CgA), cytokeratin (CK), S-100, P53. The level of testosterone decreased to 15.8 ng/dl on the 3rd day after operation, and the symptoms of virilization were alleviated during the follow-up, which further confirms the adrenal etiology of the testosterone production.

## Discussion and conclusions

The postmenopausal female with elevated androgens may present with symptoms of hirsutism and virilization. Hirsutism is defined as excessive terminal hair that appears in a male pattern. Virilization includes balding, deepening of the voice, cystic acne and clitoromegaly. The main androgens in women are testosterone, dihydrotestosterone, DHEA, DHEAS and androstenedione. Among them, testosterone is the most active and also plays an important physiological role. One fourth of testosterone is synthetized in the adrenal gland, one fourth in the ovaries and a half produced from the peripheral conversion of their precursors (androstenedione, DHEAS, DHEA) [2]. The major androgens secreted by adrenal cortex are DHEA and its sulfate (DHEAS), and androstenedione while only a small amount of testosterone was directly synthesized by adrenal cortex [5]. Therefore, the increase of serum DHEA, DHEAS, androstenedione is generally regarded as adrenal origin, and isolated elevated testosterone is usually thought to originate from the ovary in women with hyperandrogenemia [6]. The main differential diagnosis of hyperandrogenemia is either tumoral causes, namely androgen-secreting ovarian or adrenal tumors, or non-tumoral causes, such as polycystic ovarian syndrome, Cushing’s syndrome, congenital adrenal hyperplasia, and iatrogenesis. The postmenopausal woman with rapid progression of clinical symptoms or highly elevated testosterone levels should be suspected of androgen-producing tumors [1, 7]. In postmenopausal woman, ovarian causes of hirsutism and virilization are more common than adrenal origin, while the adrenal etiology of androgen production was confirmed in our case. Androgen-secreting adrenal tumors, most of which co-secrete androgen and cortisol, are rare in clinical practice and often diagnosed as adrenocortical carcinoma, while pure androgen-secreting adrenal tumors (PASATs) are more rarely. Moreno et al. [8] reviewed 801 adrenalectomies from 1970 to 2003, only 21 cases were due to PASATs, while all these PASATs had elevated testosterone and DHEAS.

It can be seen from above that PASATs are rare and usually accompanied by a concomitant increase in common adrenal androgens such as DHEA, DHEAS, and androstenedione. Our case is unusual, for that testosterone levels were within tumor range, while the serum DHEAS, androstenedione levels were normal. The imaging results, postoperative pathology and postoperative normal testosterone levels confirm the adrenal etiology of the androgen production. As to our knowledge, very few cases of PASATs that only secrete testosterone had been reported [9–12]. From these cases, we find that adrenal tumors secreting testosterone often have low malignant potential and misdiagnosed as ovarian disease due to its normal DHEAS levels. Surgical resection has a good effect on treatment of these tumors, but none of them did immunohistochemistry to confirm the nature of these tumors. In our case, immunohistochemistry showed that the tumor was positive for inhibin α, melan A, SYN (focal +), Ki-67 (< 3%), negative for CK, CgA, S-100, which supported benign adrenocortical adenoma.

The exact pathogenesis of these testosterone-secreting adrenal tumors is unclear. In healthy individuals, circulating DHEAS levels are known to reach a peak in the early 20s, and decrease linearly thereafter [13]. Carlos Morán et al. [14] found that such an age-related decrease of DHEAS levels also exists in hyperandrogenemia patients. Therefore, we speculate that DHEAS levels may tend to be not high in elder women with PASATs. Aguirre [15] suggested that testosterone-only adrenal tumors may actually originate from translocated gonadal cells. From 1981 to the present, to our knowledge, a total of 4 cases of adrenal adenoma secreting androgen were reported to have been found the specific Reinke crystallization of Leydig cells, suggesting the possibility of differentiation into Leydig cells during the process of tumorigenesis and development of tumor cells [11, 16–18]. In this case, we didn’t find Reinke crystallization. However, the absence of these inclusions does not exclude a Leydig cell character, because those inclusions are present in only 40% of Leydig cell tumors and the immunohistochemistry of our patient revealed that the tumor was positive for inhibin α. Inhibin α was a commonly used immunohistochemical index for diagnosis of Leydig’s cell tumor, even the specificity of inhibin α is limited since it is also expressed in adrenal cortical tumors. Besides, it was revealed that β-catenin over-expression might be also involved in the tumorigenesis of these pure testosterone-secreting adrenal adenomas.

When hirsutism accompanied by virilization signs such as severe balding and clitoromegaly appear in postmenopausal women, an underlying androgen-secreting tumor should be suspected. Adrenal tumors secreting only testosterone with no concomitant increase of common adrenal androgens such as DHEA, DHEAS, and androstenedione are extremely rare. This case reports an unusual case of a postmenopausal woman with a pure testosterone-secreting adrenal adenoma and shows that in the daily clinic, the source identification of hyperandrogenemia only based on the levels of testosterone, DHEAS and androstenedione is limited. The suspicious patients are recommended to receive a thorough pelvic examination and adrenal imaging examinations. The testosterone secreting tumors are able to induce severe hyperandrogenism and virilization, and laparoscopic resection provides a very effective treatment. PET-CT is helpful in differentiating benign or malignant testosterone-secreting adrenal tumors and excluding ectopic tumors.

In conclusion, our case indicates the importance to evaluate not only ovaries but also adrenals in all women with virilization particularly during menopause, even their androstenedione, DHEA and DHEAS level are normal.

## Abbreviations

ACTH: Adrenocorticotropic hormone
CgA: Chromogranin
CK: Cytokeratin
DHEA: Dehydroepiandrosterone
DHEAS: Dehydroepiandrosterone sulfate
hCG: Human chorionic gonadotropin
MRI: Magnetic resonance image
PASATs: Pure androgen-secreting adrenal tumors
SUV: Standard uptake values
TSH: Thyroid- stimulating hormone
